# ‘Between-time stories’: waiting, war and the temporalities of care

**DOI:** 10.1136/medhum-2019-011810

**Published:** 2020-04-27

**Authors:** Laura Salisbury

**Affiliations:** Department of English and Film, University of Exeter, Exeter EX4 4QJ, UK

**Keywords:** literature and medicine, psychoanalysis, psychiatry, medical humanities

## Abstract

This article analyses how World War II shifted and contained embodied experiences of waiting in relation to broader ideas of lived time in modernity. The trench warfare of World War I has often been imagined as a limit experience of anxious waiting, but World War II produced compelling accounts of experiences of suspended time in civilian populations exposed to the threat and anticipation of ‘total war’. This article analyses representations of this suspended present drawn from Elizabeth Bowen and Virginia Woolf, alongside materials in the Mass Observation Archive, to develop an account of how exposure to a future shaped by the potential of annihilation from the air reshaped experiences of durational temporality and the timescapes of modernity in the London Blitz. It also explores the relationship between anxiety, waiting, and care by attending to psychoanalytic theories that developed in the wartime work of Wilfred Bion and Melanie Klein. Extending Freud’s account of anxiety as producing ‘yet time’, this article describes how and why both literary and psychoanalytic texts came to understand waiting and thinking with others as creating the conditions for taking care of the future.

At some point in 1940, during a period 57 days of consecutive bombing, a woman and a man are imagined walking together in London:

Full moonlight drenched the city and searched it; there was not a niche left to stand in. The effect was remorseless: London looked like the moon’s capital—shallow, cratered, extinct. […]

However, the sky, in whose glassiness floated no clouds but only opaque balloons, remained glassy-silent. The Germans no longer came by full moon. Something more immaterial seemed to threaten, and to be keeping people at home. This day between days, this extra tax, was perhaps more than senses and nerves could bear.1

First published in 1944, when the V1s and V2s were now flying overhead, Elizabeth Bowen’s ‘Mysterious Kôr’ takes a ‘shallow, cratered, extinct’ lunar landscape more commonly associated with World War I and transports it to civilian London. The young woman, Pepita, is walking with her soldier lover, Arthur, and suddenly imagines another brittle bright, uncanny place:

​‘Mysterious Kôr thy walls forsaken stand

​The lonely towers beneath the lonely moon—’

Pepita goes on, describing Kôr as ‘a completely forsaken city, as high as cliffs and as white as bones, with no history’.2 But she then muses: ‘If you can blow whole places out of existence you can blow whole places into it’.3 She imagines Kôr as somewhere she and Arthur might live and be alone, instead of the pinched ‘flatlet’ Pepita shares with a girl called Callie to which they are about to return. Another space in which bodies might be able to subsist and express their desires gets blown into Pepita’s mind by the Luftwaffe. But if Kôr is another kind of place, the hallucinated ‘timeless city’ also represents a new temporality that emerges from this suspended time of aerial bombardment. This article sets out to explore the qualities and social life of this waiting time, suggesting that the ‘day within days’ blown into existence by the lived realities of 20th century industrialised war both tracks the timescapes of modernity and is significantly illuminated by the demands and impossibilities of care such times evoked. By paying particular attention to anxious temporal experiences in literary and psychoanalytic texts of World War II, it examines how waiting and thinking with others came to be understood as creating conditions for taking care of the future.

## Yet time

The poem Bowen has Pepita half-remember is ‘She’ by Andrew Lang. Published in 1888, it is dedicated to H. Rider Haggard, the author of the first novel in the ‘lost world’ subgenre of scientific romance: *She: A History of Adventure* (1886–87). *She* was an extraordinarily popular piece of imperial adventure fiction concerning a lost, ruined city called Kôr in Africa and a white queen, Ayesha, who has been waiting more than two millennia for her reincarnated lover. In a 1947 radio broadcast, Bowen spoke of the thrill of first reading *She* as a bored 12-year old and being prepared thereafter ‘to handle any book like a bomb’4—ready for the incendiary qualities of fiction capable of exploding creativity in the waiting adolescent mind. But what strikes Pepita is that both novel and poem were written ‘when they thought they had got everything taped, because the whole world had been explored, even the middle of Africa’. And this leads to her assertion that ‘*[t]he world is disenchanted*’5—a quotation from Lang’s poem that is linked, through Schiller, to the Romanticism of a pagan world lost and past, but which goes on to resonate through the 20th century via Max Weber’s articulation in 1917 that in modernity ‘there are no mysterious incalculable forces that come into play […] one can, in principle, master all things by calculation. This means that the world is disenchanted’.6 Both Lang’s poem and Haggard’s novel address the Victorian sense that the final spatial frontier had been reached. This sense of boundedness led to scientific romances opening up new worlds beyond land-locked geographies by crossing temporal frontiers into a time in which positivist historical progress has been radically suspended.7 But the ‘enchanted’ world that Pepita finds in Kôr, like the one Bowen finds in the supernatural genre, is not an evasion of modernity, of history. Instead, it slips into view in the uncanny places and times historical modernity produces: in new communication technologies like telephones;8 in the city under aerial bombardment; in the air raid shelter; in the reconfigured Victorian drawing-rooms carved into cramped flats with paper-thin walls.

At one level Kôr is simply an escape: a fantasy space outside of the dreary, straitened conditions of wartime London where Pepita and Arthur do not have to wait be alone. Kôr is also a suspended moment where the passage of time does not bring the lovers any closer to their deaths. But it is also clear that the fantasy of Kôr, which tricks its way into consciousness through war, also tracks particular modes of temporal extension that war produced, as people drifted ‘afloat on the tideless, hypnotic, futureless to-day’.9 Bowen’s short story certainly takes place in an interval that seems out of time. As she writes in her US postscript to *The Demon Lover* collection, these short story fantasies are ‘by-products of the non-impulsive major routine of war. These are between-time stories—mostly reactions from, or intermissions between, major events. They show a levelled-down time’.10 Indeed, almost nothing happens in ‘Mysterious Kôr’ in terms of narrative action. Arthur and Pepita walk and return to the flat where Callie has given up waiting for them and fallen asleep. Callie appears like votive candle—‘sedate, waxy and tall’—with her virginal vigil representing another kind of waiting to contrast with Pepita’s passionate, embodied immediacy. Callie and Pepita share a bed while Arthur sleeps on the divan. Callie goes to speak to Arthur at 4am. They talk. Nothing happens. Callie goes back to bed where Pepita seems to be dreaming of Kôr. But Bowen’s adumbration of the time taken when nothing takes place, again and again, is a way of doing justice to the experience of war in London where soldiers and civilians alike faced potential annihilation from the air. As Bowen herself put it, ‘[t]here are no accounts of war action’ in these stories; they are ‘studies of climate, war-climate’.11 For the majority who came through an air raid, their continued existence signalled that a certain kind of nothing had indeed happened to them. Surviving represented both the banality of nothing occurring and a jewelled sense of fortune’s touch—in HD’s description of the aftermath of aerial bombardment, ‘[h]our is cut from hour in precious prismatic fragments […] An extra day has been given us’.12 Although anxious waiting for news of loved ones strongly characterised affective life for civilians in World War I,13 to have survived the Blitz on the home front of World War II was to have endured and perhaps temporalised internally a rather different experience of waiting: the particular psychic states elicited by drawn out nights in vulnerable houses, air raid shelters, or in the underground, waiting for bombs to drop that had not yet come for them.

The suspended animation of ‘Mysterious Kôr’ resonates with Paul Saint-Amour’s reading of the interwar period as a waiting time, ostensibly between World War I and the end of World War II, but still present in Cold War literatures and in many of the preoccupations of the present, that was dominated by a frightened anticipation of conflict to come.14 By 1932, Prime Minister Stanley Baldwin had insisted in a speech called ‘A Fear for the Future’ that the ‘man in the street’ must ‘realise that there is no power on earth that can protect him from being bombed […] the bomber will always get through’.15 According to Saint-Amour, the possibility of total war, experienced by civilians in perhaps its clearest form via the air-raid, produced a very particular cultural phenomenon: a collective anxiety about exposure and threat that shaped the possibility of the future itself. Although most people were not physically injured by bombs during the Blitz, Michal Shapira has noted the ubiquity of experiences of anxious waiting: ‘[o]n average, Londoners were threatened once every 36 hours for more than 5 years, with sirens heard on 1224 occasions’.16 And as Saint-Amour puts it, this anxious threat of destruction produced its own trauma: ‘violence anticipated is violence already unleashed’.17

Saint-Amour argues, however, that most of the cultural and critical engagement with war has concentrated on traumatic memory and on a stuck temporality concerned with unbidden repetitions of the past. And this comes at the expense of the possibility of understanding the collective experience of ‘the *eventuality* of a future-conditional war’: ‘a proleptic mass traumatisation: a *pre* traumatic stress syndrome’.18 Saint-Amour indeed reads Freud’s 1926 work on anxiety dismissively, finding only the seemingly strange idea that anxiety neuroses, even in wartime, emerge from a psychosexual ‘historical factor’ reactivated in the present to produce a traumatised response.19 But although we may be afraid of things we know to be dangerous, in her more sympathetic account of Freud’s thinking Lynsey Stonebridge explains that ‘we are not actually anxious about the things we know’; instead, anxiety neurosis ‘tells the affective history of a life that is not consciously known—the life of the unconscious and of fantasy, […] the traumatic childhood of the real dangers of the present moment’.20 In Freud’s view, as with all symptoms, anxiety neurosis is produced in relation to a ‘danger situation’ (either external or internal) as an attempt to manage in the present a psychic disequilibrium emergent from a reaction to a possible future, but determined by a traumatic structure laid down in the past. Anxiety, like waiting, is always orientated towards a future; but the capacity to tolerate the affects produced by these dependent relationships towards the future are determined by one’s expectations of what it might bring. Will it deliver something that will have been worth the wait, or a repetition of deprivation or violence? The quality of expectation is necessarily drawn from experience in the past, but the practice of waiting requires imagining and enduring in the present at least the possibility of a future where things would be different.

Although Saint-Amour insists that ‘Freud’s metapsychology remains fixated on the subject’s primordial past, recollected or repressed’,21 Freud is less definitively turned towards the past than he implies; rather, as Stonebridge puts it, anxiety looks ‘both forwards and backwards’.22 Waiting for something that evokes the possibility of annihilation and a breaking apart the psyche cannot imagine itself surviving will likely produce anxiety automatically. But Freud’s more counterintuitive claim is that anxiety also functions as a signal that enables defences to be mobilised: the individual ‘can foresee and expect a traumatic situation of this kind which entails helplessness, instead of simply waiting for it to happen’. Freud writes of signal anxiety:

‘the present situation reminds me of one of the traumatic experiences I have had before. Therefore I will anticipate the trauma and behave as though it had already come, while there is yet time to turn it aside’. Anxiety is […] on the one hand an expectation of trauma, and on the other a repetition of it in a mitigated form.23

Anxiety, here, takes a certain care of the self, just as an air raid siren is a warning that gives time for defence: signal anxiety mitigates helplessness by enabling the psyche to feel that ‘there is yet time’ to act and perhaps wait in a safer, more sheltered place. Waiting produces anxiety, but the neurotic repetitions and obsessive compulsions of anxiety also seem aimed at producing a world in which there would be time enough to wait.

Saint-Amour argues, surely correctly, that anxious waiting becomes one of the dominant affects of a 20th century subjected to two global conflicts and the threat of total war via aerial bombardment. Bowen’s ‘Mysterious Kôr’ certainly starts by imagining itself airborne: ‘from the sky, presumably, you could see every slate in the roofs, every whited kerb, every contour of the naked winter flowerbeds in the park’.24 But this story gestures beyond the affect of anxious anticipation by evoking another kind of waiting that links its ‘day within days’ to the sense that there could be ‘yet time’ that anxiety seems to promise. As Lisa Baraitser has argued in relation to the contemporary moment of late liberalism, when the spooling of time towards a possible future comes unravelled, experiences of interruption, suspension, delay and slowness strongly insist in affective life, often enduring in and as traditionally feminised practices of care.25 To speak of practices of care is to describe what Joan Tronto names as ‘everything that we do to maintain, continue, and repair our “world” so that we can live in it as well as possible’.26 And although such practices seem opposed to the trajectory of mobilisation that putatively underpinned military time, just as the contemporary moment seems allergic to the time of sustaining and enduring, the ‘non-impulsive major routine’ blown into existence by the threat of a cancelled future was a time shot through with both waiting and care.

While we might be anxious in relation to what we care about, ‘care’ has a semantic doubleness that maintains a particular conceptual link between making provision and worry. To take care is to look after, to give cautious attention; but the very oldest usages of care link it to the Old High German *chara*: grief, lament and mental suffering.27 This linguistic proximity perhaps helps us understand something of the peculiar mirroring of the repetitive returns of an anxiety neurosis that produce a sensation of ‘yet time’ and a future, and the sustained attention and skilled anticipation of care time that ‘looks after’—making the possibility of a future in the present, even if only a minimal one. Maria Puig de la Bellacasa has recently described how care involves ‘a lot of hovering and adjusting to the temporal exigencies of the cared for’;28 and here the vigil of care and repetitions of reassurance seem not so far from the obsessive returns and hypervigilance of anxiety. But while care clearly takes time, it also produces an awareness of a temporisation of experience that resists the grain of master narratives of future. Puig de la Bellacasa suggests that care time runs counter to the temporality of anxiety, however: ‘with regard to anxious futurity, feelings of emergency and fear, as well as temporal projections, need often to be set aside in order to focus and get on with the tasks necessary to everyday caring maintenance’.29 Certainly, fear of the future needs to be wound back while focusing in the present on the needs of others and everyday practices of endurance. But the repetitions of care, in ‘looking after’, nevertheless retain a certain, weakened commitment to a future—an ‘after’ into which selves and others might at least minimally endure—that is perhaps not to be fully separated from the ‘yet time’ signal anxiety looks to produce. In what follows, I want to outline the ways in which a collective awareness in military and civilian populations of threat that could not be easily fought but only endured—the awareness of ‘total war’—became crucially entangled with both the suspensive temporalities of modernity and the practices of care such waiting times demanded and induced.

## Waiting machines

If, as Ghassen Hage has suggested, experiences of waiting are never just existential but are always historically articulated,30 what does it mean to wait in a ‘disenchanted world’? Reinhart Koselleck has demonstrated that in Western Christian cultures a linear time of expectation orientated towards the Second Coming of Christ persisted into 16th century.31 In Benedictine monastic life, for example, a daily temporal ‘culture of vigilance’ was explicitly structured to meet St Paul’s demand for ‘constant prayer’ while awaiting the Second Coming,32 while in much pre-industrial or para industrial life waiting could be gathered up into more general cyclical rhythms and practices of activity as it played its part in contingent but imaginable returns. But as waiting for God as the final backstop for human experiences of endurance and unfulfillment became less culturally available in many Western cultures of modernity, there was a waning of that sense of final reckoning at the end of the ‘end times’ that gave waiting its eschatological value and its *for* a structuring quality. As Koselleck has shown, the timescapes of Western modernity decisively shifted as increasing secularisation emphasised human political and economic activity shaping the conditions of an unknown future.33 Indeed, by the 19th century, waiting was more clearly associated with the redundancies built into standardised or industrialised time regimes tabled according to the needs of modernity. As is well known, the advent of the paired systems of railway and telegraph wire both required and enabled national time regimes.34 But while mechanisation encouraged the tabulation of time and its equation with productivity—as time became standardised, more easily quantifiable, more susceptible to being cut up into blocks that could then be exchanged as units—there emerged a sense that temporality might more authentically be found within subjective experience, in which time’s felt pace of passing became the essential mode of measure. As Henri Bergson affirmed in 1907, ‘all our operations on the systems that science isolates, rest in fact on the idea that time does not bite into them’.35 But in his account of waiting for sugar to dissolve in water, Bergson articulates how the movements and qualities of matter cannot be separated from the duration of consciousness that perceives them. ‘[T]he time I have to wait is not that mathematical time’; rather, waiting time ‘coincides with my impatience, that is to say, with a certain portion of my own durations, which I cannot protract or contract as I like. It is no longer something *thought*, it is something *lived’*.36 For Bergson, the lived experience of duration—the essential quality of temporality that stood outside of positivist accounts of quantifiable time—obtrudes for philosophical reflection precisely in the moments it resists being seized and rationalised.

Anson Rabinbach has argued that World War I put an end to many of the agonised debates about Taylorism in Europe and the scientific management of time.37 Governments sacrificed new social policies on overwork, health requirements, and the protection of women and children in the workplace under the demands of industrialised warfare that simply could not wait. But war clearly did not only produce the efficient use of time, just as the lived experience of global industrialised conflict came to splinter the imagined ladder of positivist historical progress. Henri Barbusse’s *Under Fire* (1916)—one of the first extended literary accounts of life in the trenches—describes humans turned into the equipment of war:

We are waiting. We get tired of sitting down, so we get up. Our joints stretch with creaking sounds, like warped wood or old hinges: damp rusts a man as it does a rifle, more slowly, but more profoundly. And we start to wait again, differently.

You are always waiting, in wartime. We have become machines for waiting.

For the moment what we are waiting for is grub. Then it will be letters. But everything in its own time: we’ll think about the letters when we’ve finished the meal. Then we’ll start to wait for something else…38

Imagined as mechanical equipment, the *for* in the soldiers’ waiting is endlessly substitutable, endlessly fungible. In the face of the impossibility of finding a place in a narrative of progress or, more pressingly, of bringing to consciousness the reality of what might imminently await them, Barbusse speaks of men transformed into ‘machines for waiting’ in which, at least at first glance, all time seems ‘occupied’ or seized, without any time being available.

Of course, to endure and experience becoming a waiting machine, to experience falling out of sync with standardised time, is precisely what mechanisms do not and cannot do. And for the phenomenological psychiatrist Eugène Minkowski who, unlike Freud, saw action in World War I, this disruption of duration, which Kate McLoughlin suggests is a precipitate of combat experience,39 defines the contours of mental illness itself. For Minkowski, mental illness almost always entails a perception of being detached from the temporality he represents as comfortable to human life: a ‘lived synchronism that we expect to find in the general feeling of moving with time and in step with it’.40 In a chapter of *Lived Time* drafted just after Armistice, Minkowski reimagines waiting not as boredom but as entwined with the nameless dread of what cannot be seized. He writes positively of ‘activity’, but ‘expectation’ is different:

[it] englobes the whole living being, suspends his activity, and fixes him anguished, in expectation. It contains a factor of brutal arrest and renders the individual breathless. One might say that the whole of becoming concentrated outside of the individual swoops down in a powerful and hostile mass, attempting to annihilate him; it is like an iceberg surging abruptly in front of the prow of a ship, which in an instant will smash fatally against it. Expectation penetrates the individual to his core, fills him with terror before this unknown and unexpected mass, which will engulf him in an instant […] In the presence of an imminent danger we wait, frozen in place as if paralyzed by terror. 41

When describing a Russian soldier traumatised by war, Minkowski anatomises the temporal distortions experienced by the person incapable of inhabiting an active relationship towards the future. This sense of time does not simply match the heightened affect of anxiety with the a future experienced as a hostile, destructive force, however; anxiety starts instead to shudder into depressed affect. As the solider himself states: ‘I feel time flee, but I don’t have the sensation of following the movement; I have the feeling of turning in the opposite direction than the earth’.42

Although the Russian soldier may be reacting to his combat experience, Minkowski suggests that the psychic logic of war becomes totalised when the future comes to feel cancelled and the *for* of one’s waiting is lost, arrested, or imagined as a repetition of the present saturated with the traumas of the past. To think back to Freud, the symptom may make an attempt to restore psychic equilibrium by producing a feeling that ‘there is yet time’, but in doing so it creates an over-abundance of time that does not pass. But if anxiety shades into depressed affect, depression can, in turn, shudder back into anxiety: Minkowski observes that ‘[w]hen the flow of time is only slowed down, it is the obsessive phenomena that intrude’.43 Indeed, he notes that the patient may attempt to control and reinstate a ‘mechanical progression’ of time through obsessive symptoms that give rise to an illusion of forward movement, but only fill the present by filling in for time’s ‘weakening dynamism’.44

The anxious sensation that ‘there is yet time’ can, of course, be transmuted into a hermetically sealed fantasy, just as Minkowski’s soldier feels he is the only person on earth rotating otherwise. Pepita ends up in her solitary fantasy space and time alone, without Arthur: ‘He was the password, but not the answer: it was to Kôr’s finality that she turned’.45 But waiting time might also produce other possibilities for experience. Bowen’s readers may wonder whether Pepita is looking to fall back into a ‘white time’ of the Imperial past,46 or if she is searching out a spectral, ultimately unwritten women’s or even queer time in Kôr and in ‘She’. As she assertively detaches herself from Arthur and heterosexual coupling, rejecting his suggestion that they might ‘[p]opulate Kôr’ with children, one wonders what other kinds of ‘answer’ might await Pepita, even as Kôr can only be entered in a solitary way, here, rather than as a space of sociality in which a queer life might be lived.

Bowen’s accounts of the waiting time of war mostly concentrate on heterosexual erotic intensity and the strange individual freedoms lived out as a new time suddenly gaped between work and leisure—that defining timescape of industrialised modernity. This element captures Lara Feigel’s imagination as she explores how an artistic aristocracy, already somewhat liberated from modern time regimes, pursued what Graham Greene later described as a somewhat ‘manic-depressive’ mode,47 strung out between the euphoria of surviving an air-raid, reactions to threat that were depressed or anxious by turn, the erotic charge set off by the imminence of death during the Blitz, and the melancholic aftermath of postwar austerity. But if we return to *Under Fire*, written during World War I, another sense of waiting time flickers into view. There, becoming a waiting machine is not clearly anxious or arousing, although anxiety and bodily tenderness are not completely absent. ‘The frightful narrowness of communal life compresses us, adapts us and blends us into each other’,48 Barbusse writes. But in this ‘fatal contagion’ there is also a significant erosion of the distinctions between work and leisure, activity and inactivity. And Barbusse observes that in place of the ungraspable, unthinkable *for* of the squad’s waiting, certain practices of waiting *with* emerge that oddly confuse the mobilisation of the military industrial complex with domestic practices of care. Neither clearly productive nor unproductive, these minimal inclinations towards the immediate, pressing requirements of care use the time of waiting to render bearable an experience and produce a future in the face of a *for* that cannot be brought to consciousness.

Through a reading of Robert Graves’s 1915 poem ‘It’s a Queer Time’, Elizabeth Freeman suggests that World War I contains shards of a decidedly queer time of sociality that refuses ‘the foreshortened time of the patriot or the eschatological scheme where death leads directly to salvation’. Instead, the poem ‘narrates military history’s failure to fully organise time towards nationalist ends’.49 If certain kinds of time in modernity pull human minds and bodies towards quickening and synchronising elements of everyday existence to maximise productivity, while ‘offering up other spaces and activities as leisurely, slow, sacred, cyclical’, the soldier in the in-betweens of time regimes finds himself accessing ‘a time whose violence offers up queer possibilities despite itself’.50 This queer time, which is also a profoundly demobilised time (neither clearly productive nor obviously unproductive), is indeed both produced by war and unfolds as one of its core experiences. Violence in the past is contained, if only momentarily, and violence in the future is anticipated but suspended through acts of care that remake the world, even if only minimally and temporarily.

In Mary Borden’s clear-eyed account of nursing in World War I, we get a sense of both the relentless demand for attentiveness produced by war and the impossibility of a delivering a care that could fully meet it. In *The Forbidden Zone* (1929) Borden outlines the temporal presence and violent ruptures that come with being charged with caring for others and the unknowable future that relationships with an other invoke. As she tends the wounded with the help of orderlies she calls ‘old ones’, time seems to stop:

It is a scene in eternity, in some strange dream-hell where I am glad to be employed, where I belong, where I am happy […] We are locked together, the old ones and I, and the wounded men; we are bound together. We all feel it. We all know it. The same thing is throbbing in us, the single thing, the one life. We are one body, suffering and bleeding. It is a kind of bliss to me to feel this.51

But following this presence and contact there is the inevitable rupture of triage: “No, not that one. He can wait”.52 Borden frequently describes her nursing of bodies in domestic terms: sewing, mending, laundry. Of one man waiting under electric light bulbs she writes, blankly: ‘leave him to cook there’. She necessarily turns her mind elsewhere, stepping out of the fantasy that there is no gap between need and fulfilment, but a voice calls out:

Sister! Oh, my sister, where are you?

[…] It is the blind man calling. I had forgotten him. I had forgotten he was there. He could wait. The others could not wait. So I had left and forgotten him.

She runs to him:

You are not alone, I lied. There are many of your comrades here and I am here, and there are doctors and nurses. […]

I thought, he murmured in that faraway voice, that you had gone away and forgotten me, and that I was abandoned here alone.

My body rattled and jerked like a machine out of order. I was awake now and seemed to be breaking to pieces.53

The experience of being an oiled machine, of being ‘locked together’ that paradoxically produces a feeling of humane ‘life’, jams. Borden cowers behind a screen. The fullness of time is ruptured by the reality of the insufficiency of her care and attention and the violence as she is forced, by the brute presence of finite bodies and a production line of injuries, to drop the fantasy of a perfect fit between need and resources that would allow her to wait fully with these men. In the face of this rupture of care, however, a small repair is offered back—another domestic gesture—that is minimally inclined towards a future that can only be meagrely inhabited, but that exists nevertheless. In a gesture of making do, of all one can do, one of the old orderlies sticks his grizzled head around the corner of the screen: ‘He held his tin cup in his hands. It was full of hot coffee. He held it out, offering it to me. He didn’t know of anything else he could do for me’.54

## Demobilised time

In her postscript to the *The Demon Lover* (1945), Elizabeth Bowen wrote that ‘[l]iterature of the Resistance has been steadily coming in from France. I wonder whether in a sense all wartime writing is not resistance writing’. She noted, however, that such resistance was not necessarily martial or political; rather, ‘[p]ersonal life here, too, put up its own resistance to the annihilation that was threatening it’. Although Bowen insistently emphasises ‘individual destiny’,55 as in Pepita’s solitary fantasy, clear attention to the intersubjective qualities of waiting time can be found in a ‘Resistance’ play from 1953 that has its own relationship to war and occupation. Samuel Beckett’s *Waiting for Godot* certainly implies a metaphysics of temporality and waiting’s place within that; but criticism has come to understand *Godot* as linked both to Beckett’s experiences in the French Resistance and to the affects of waiting in a socio-historical moment in which the promise of the future has sheared away.56 Denuded of any sense of progress or a future that might be seized, *Godot* evokes instead a present that is queerly occupied. Neither weak enough to cease and desist, nor strong enough to leave, Vladimir and Estragon stand, or sit, and wait; they endure and persist, not really waiting for Godot, but waiting *with*, and sometimes even *on* one another. Keeping occupied, ‘passing the time’ together in games of give and take, of violence and of care, indeed becomes the quality of this time without qualities. In Beckett’s 1957 *Endgame*, interpretable as both a displaced account of World War II and an anticipation of a nuclear Holocaust that would reach beyond the ruined cities of Hiroshima and Nagasaki, Hamm cannot stand and Clov cannot sit. Clov tells Hamm there is no more painkiller; indeed, all the things they may have once been waiting for have been exhausted. The alarm clock does not work anymore, and yet the time that time takes to come to an end remains as the characters endure in an extenuated yet nevertheless finite state. They are dependent; they are ‘obliged to each other’.57 Indeed, as Barbusse and Beckett both show, where people wait alongside others social practices of waiting *with* seem to emerge.

As is well known,58 in the mid 1930s, Samuel Beckett sought psychotherapy for what would now likely be diagnosed as an anxiety disorder. When Beckett met his psychotherapist Wilfred Bion in 1934, there was much in their social background that was shared; but Beckett understood that one key experience was not. Bion was born in 1897 and just 9 years older than Beckett, but, like Beckett’s friend the poet Thomas McGreevy, Bion had survived Ypres; as a tank commander in World War I he had also won the Distinguished Service Order for ‘conspicuous gallantry and devotion to duty’. Beckett wrote to McGreevy telling him that he experienced Bion ‘against a background of Toc H and Tank Corps that makes me tremble’.59 He could hardly have known then how his own experiences in the French Resistance in World War II, so decisive in turning him into the figure we now recognise *as* Beckett, might bring his work into peculiar proximity to the later Bion’s psychoanalytic commitment to slow process and practices of waiting.

In 1938. Bion began a training analysis with John Rickman, but in 1940 was recommissioned into the Royal Army Medical Corps. And it is clearly in the wake of one war and in anxious anticipation a second that Bion wrote his first published paper, ‘The “War of Nerves”: Civilian Reaction, Morale and Prophylaxis’. Here, Bion addresses the dangers for civilians of anxious waiting in relation to the anticipated violence of ‘intensive totalitarian war’.60 Suggestively, Bion’s essay was likely written up during the waiting time known as the ‘phoney war’ when there were no major military land operations on the Western Front and no civilian experience of aerial bombardment. The essay followed the convening of a voluntary committee of medical officers, army psychiatrists, and members of the Tavistock Clinic in 1938, who were concerned with the inevitability of a devastating air-attack on London, the likelihood of civilian panic, and the potential for an ‘epidemic of shell-shock’ comparable ‘to that observed during the war of 1914–18’.61 In his essay, Bion argues that an enemy can wage war by arousing infantile emotions of fear and dread in order to distract the population from the conflict being fought in external reality. For civilians during the time of the phoney war, Bion notes that ‘the field is held by *phantasies* of air-raids’, with this form of attack represented as the very ‘opposite of a psychotherapeutic procedure in which the knowledge of the unconscious on the part of one participant is used for the benefit of another’.62 Bion’s essay is thus concerned with developing a form of ‘psychological A.R.P.’63 (air-raid precautions) as a practice of care for a civilian population newly exposed to military conflict. Bion asserts that they must not be left to languish in a kind of waiting time (what Minkowski would call ‘expectation’) in which phantasy can take hold. Instead, as soon as an air-raid siren goes off, ‘[t]he alarm […] must be a call to action, and there must be an action to which every man and woman is called’.64 Solitary, isolated and isolating waiting, which can lead to neurosis and what the later Bion invokes as ‘not a fear of dying made tolerable, but a nameless dread’, must be replaced with communal effort and action towards a graspable, survivable future.65

Bion had learnt from personal experience how standard temporalities unravel during war. As he put it in the posthumously published *The Long Week-end*, even when only on the way to the Front, ‘time had already come to mean something that could not be measured by watches’.66 Although war was clearly full of events and the most profound anxieties, in the face of a cancelled or unimaginable future, thinking and feeling and their relationship to life and the events of history paradoxically began to leak away:

We stood there and waited for something to happen. We had not even begun to realise that nothing happens in war, or – which comes to the same – nobody knows what happens. I would have thought I was being made a fool of if I had been told that, even years after the war and another like it, I still would not know something so simple and obvious as who had won.67

Something happens, but it cannot be mobilised in the realm of knowledge. Bion describes the experience of combat itself sometimes producing a manic ‘dis-association, de-personalisation [t]hat was a way of achieving security—spontaneous, automatic, but potentially costly as it involved not knowing of the imminence of death’.68 Alongside this dangerous dissociation, he recalled, over-again in shame-pocked prose accounts, a terrible evacuation: a moment on the Amiens-Roye road when the 20-year-old Bion found himself unable to tolerate waiting with a dying young runner, Sweeting, whose chest had been blown apart. ‘Mother, Mother, write to my mother, sir won’t you? … Mother, Mother, Mother’, implores the ‘boy’. The terrified, vomiting Bion shouts: ‘Oh for Christ’s sake shut up’.69 For Bion, the carapace of military camaraderie before and after such events was a profoundly inadequate defence in the face of such extremity. He writes elsewhere that during the war he often ‘felt that all anxiety had become too much; I felt just like a small child that has had a tearful day and wants to be put to bed by its mother; I felt curiously eased by lying down on the bank by the side of the road, just as if I was lying peacefully in someone’s arms’,70 although he was not to develop his thinking about how such catastrophic fears might be absorbed, contained, and understood, rather than evaded in fantasy or vomited out, for decades. Barbusse, however, was able simply to note the minimal emergence of certain kinds of waiting *with* that sat alongside an ungraspable, unthinkable *for* that oddly confused the mobilisation of the military industrial complex with domestic social practices. Here, time was held and contained, not exactly through the practical, futural activity Bion had recommended for civilians in 1940, but through certain social, para-domestic practices of waiting—a being present with.

Significantly, the British civilian population did not, in general, collapse in the face of aerial bombardment in the way Bion feared it might; instead, often people just waited it out, living on and getting on, with more or less success, through daily practices in which the temporal relationship to the future and unbearable anxiety was endured, borne, and contained within minor, domestic modes of care, without being seized or transformed. The Mass Observation project gives some insights into these practices.71 Diarist 5278, a female, single civil servant born in 1912, records her experiences in the air-raids of August 1940. Initially, there seems to be a lack of contact with reality and its dangers as she refuses to enter the nearest air-raid shelter, stops to post a Mass Observation response, and answers back to an angry A.R.P. warden. Later, in the shelter, she writes of sitting communally with others, listening to them ‘nagging about broken social engagements and meals waiting’: ‘Atmosphere cheerful: people pull faces and say “Pretty bad” and repeat rumours and facts and wonder when the next one’s coming. At Dad’s works they had a sweepstake, by quarters of an hour, as to when the next air raid would come’.72 There is psychological defence in the domestication of fears of annihilation into the ‘Pretty bad’, and the reshaping of the imminence of death, in which the gamble of living becomes explicit, into the banal risk of a bet in which the future itself is not one’s stake. But we also see the ‘hovering and adjusting’ that Puig de la Bellacasa suggests is characteristic of ‘care time’, in which time is ‘levelled down’, as Bowen has it—flattened into minimal articulations of a future that comes to be held between people.

But if this waiting becomes attentive to what is proximate and present, it also opens up time for existential consideration. As Bowen says ‘[w]alls went down; and we felt, if not knew, each other. We all lived in a state of lucid abnormality’.73 Diarist 5278 indeed becomes reflective:

Strong feeling about everything, that it may be the last of its kind, so have (to) enjoy it. Was grateful for the moon, grateful for the sunny morning, grateful for a seat in the train, grateful for a comfortable body, grateful for being able to shut my eyes, & occasionally shut my mind, to present difficulties. Came over my mind that that’s escapism. I bolster up my self esteem by reminding myself that I’ve worried and nagged and bored all my friends, since 1933 until Munich, with talking about Nazism and war and the menace to freedom: went to meetings, read books, converted Conservatives. So when the war came, I leaned back & let my mind wander […]74

The question of what happened to people’s thinking in this ‘lucid abnormality’, of what could be contained within the psychological A.R.P. of communal action or what took place as minds began to wander, was of acute interest to British psychoanalysis. As Stonebridge writes, ‘any standard history of the Blitz can tell us that early fears of national panic and civilian air-raid psychiatric casualties were over-exaggerated, […] only one genuine case of “bomb neurosis” was reported to the British Psycho-Analytic Society’. Indeed, as she goes on to note, by 1942 the ‘Mass-Neurosis Myth’ was replaced by a ‘No-Neurosis Myth’, the psychoanalyst Edward Glover complained.75 Of course, the reality was more complex and variegated, as Edgar Jones has demonstrated in his work on the traumatic neuroses of the World War II. For although admissions to psychiatric hospitals went down during the war, the prevalence of seemingly psychogenic gastrointestinal complaints soared as digesting and processing anxious experience did not repeat the culturally overdetermined and still stigmatised symptoms of the shell shock of World War I; rather, they worked themselves through a nervous gut.76

Psychoanalysis also remained concerned and stimulated by what the war revealed about the time of psychic life, alongside the psychic life of time. In 1942, Bion and John Rickman set up the first Northfield experiment in what was the Northfield Military Hospital in Birmingham. Bion and Rickman were in charge of the training and rehabilitation wing. Their aim, ostensibly, was to improve morale by creating a ‘good group spirit’.77 To this end, Bion instructed the men to do an hour’s exercise every day and join a group.78 While this looked like traditional occupational therapy, the real therapy was the struggle to manage the interpersonal strain of groups organising things together and submitting to the dependence of sociality. Bion indeed used this experiment with groups to understand how powerful unarticulated beliefs, phantasies, and what he called, after Melanie Klein, ‘projective identifications’, could cement people within identities that enabled complicity and the potential for atrocity.

Bion went on to formulate a fundamental distinction between ‘work groups’ and ‘basic assumption groups’. As he outlines in *Experiences in Groups* in 1959, basic assumption groups are governed by a ‘proto-mental system in which physical and mental activity are relatively undifferentiated’.79 The entry into such groups is ‘instantaneous, inevitable and instinctive’,80 and there is a tendency to lose contact with external reality and act out primitive and primary processes of mind, such as splitting people and acts into good and bad objects, to use Klein’s terms. As Daniel Pick explains, ‘in the world of “basic assumptions”, thought is a misnomer, and the communications at stake are better understood as un-reflexive forms of *action*’.81 Bion indeed writes of the ‘basic-assumption mentality’ that ‘[t]ime plays no part in it; it is a dimension of mental function that is not recognised; consequently all activities that require an awareness of time are imperfectly comprehended and tend to arouse feelings of persecution’.82 In thrall to the immediacy of instantaneous, instinctive action rather than the passage of time that produces the ground for thinking, such groups find it hard to learn, acknowledge guilt and admit others into their cohort. The ‘work group’, however, can hold to the space of time of thinking that enables a relationship between, rather than a confusion of, internal and external reality; the ‘work group’ can form an alliance for thinking that can suspend action until it is thinking’s precipitate, rather than its substitute.

To explain this further we need to turn to the influential set of ideas Bion went on to develop about psychic violence and the value of psychoanalysis in a postwar world. Following his work with John Rickman, Bion undertook a training analysis with Klein between 1946 and 1952, and her work was key in developing his understanding of the link between aggression, violence and the evacuation of thinking. Perhaps strangely, instead of understanding thoughts as produced by thinking, Bion suggests in a 1962 essay that thinking evolves as a capacity for absorbing and processing ‘thoughts’ otherwise experienced as intolerable. Maybe influenced by the insistence of gastrointestinal complaints in the patients he saw or his own emetic experience on the Amiens-Roye road in 1918, Bion made a link between ‘digesting’ and the processes of thinking. For Bion, most incidences of psychopathology are precisely structured by a ‘breakdown in the development of the apparatus for “thinking”’, where thoughts run amok.83 The aim of psychoanalysis is thus for analyst and analysand to suspend the action that risks evacuating ‘thoughts’ experienced as contaminating or lacerating to the self, and instead to contain and absorb—to digest—them over time and within psychical understanding, as analyst processes and gives back thoughts as material to think with. Even decades after the war, thoughts are described by Bion via an imaginary defined by modern conflict; they are ‘evacuated at high speed as missiles to annihilate space’.84 For Bion, the only way to transform thoughts as an aerial bombardment to be managed through what he calls ‘evasion by evacuation’,85 is to suspend this mobilisation that seeks to rid of the psyche of thoughts. Instead, analyst and patient must learn together how to wait and to think. For Bion, if thoughts are missiles, genuine thinking becomes a space of embodied containment that allows the world to be taken into the self, absorbed and digested over and through time, rather than evacuated as violently invasive. For Bion, such thinking, imagined according to the processes of a body able to absorb rather than be blown apart by bullets and bombs, indeed produces a space and time where violence can be held and thought about rather than enacted. For the later Bion, alongside the practical benefits of encouraging communal, collective action to contain the anxiety of waiting time, waiting and thinking with others becomes a ‘shelter’ for the experience of time that is perhaps the only authentic psychological A.R.P.

## Thinking time

Virginia Woolf’s essay, ‘Thoughts on Peace in an Air Raid’, was published in October 1940, but it gestated just a little earlier, during the final weeks of the Battle of Britain and on the eve of the London Blitz. Woolf writes, there, of what waiting for a bomb to drop might do to thinking:

It is a queer experience, lying in the dark and listening to the zoom of a hornet which may at any moment sting you to death. It is a sound that interrupts cool and consecutive thinking about peace. Yet it is a sound—far more than prayers and anthems—that should compel one to think about peace. Unless we can think peace into existence we—not this one body in this one bed but millions of bodies yet to be born—will lie in the same darkness and hear the same death rattle overhead. Let us think what we can do to create the only efficient air-raid shelter while the guns on the hill go pop pop pop and the searchlights finger the clouds and now and then, sometimes close at hand, sometimes far away a bomb drops.86

Thoughts buzz like planes and bombs but, for Woolf, the suspended time of waiting for a bomb to drop also produces a demand for thinking that, in Bion’s terms, might effect a containment of unbearable anxiety and function, perhaps, as ‘the only efficient air-raid shelter’. For she asserts that soldiers and civilians alike are subjected to a ‘subconscious Hitlerism’, and, Woolf argues, what women might do in this moment of suspension, of apprehension, is to create an alternative to catastrophe through their capacity for thinking.

In the June of 1940, Melanie Klein was also exploring her patients’ experience of the external war and their ability to face it by linking it with their internal phantasies. She wrote in an unpublished lecture: ‘If the feeling that external war is really going on inside—that an internal Hitler is fought inside by a Hitler-like subject—predominates, then despair results. It is impossible to fight this war, because in the internal situation catastrophe is bound to be the end of it’.87 Klein also wrote extensive clinical notes on her British patients’ reactions to the *Anschluss* of Austria and Germany in March 1938 and the anxious wait, known as the Munich Crisis, before a settlement was reached in September 1938 that permitted the German annexation of Western Czechoslovakia. While Prime Minister Neville Chamberlain famously spoke of ‘peace in our time’, Klein was concerned with a more complex time of psychic life marked out by aggression and attack, shame and guilt, and by experiences in the past repeated in the present. These reflections on the ‘war inside’ were not divorced from external, political reality, however. Indeed, Klein wrote that part of their purpose was precisely to explore ‘[t]he phenomenon of whole nations submitting to dictators and being kept under by them […] We get to understand this better if we study the reactions of people who are not directly implied, but stirred in their feelings by happenings like the overrunning of Austria’.88 For both Woolf and Klein, the contours of ‘subconscious Hitlerism’ or an ‘internal Hitler’ seemed more palpable in experiences of waiting than in those of military action.

Klein was also less interested in her patients’ fears of external reality than in analysing an inner reality of primal experiences of annihilation and destructive rage that had their genesis in the childhood past, lived on in phantasy and the present of psychic experience and were brought to the fore in the face of a frightening future. In notes sent to Edward Glover she wrote that ‘[w]ith most of my patients the situation before the crisis activated deep anxiety-situations, their fears about the actual external dangers thus becoming much more acute’,89 but the analysis of these now more easily accessible internal ‘anxiety-situations’ and an understanding of their genesis in a disturbance of early relations to early ‘objects’ (usually the parents), relieved some of the external fears. When able to work through fears of a bad father (Hitler) threatening a good but vulnerable mother (for whom the Austrian, Jewish Klein frequently came to stand in), many of her patients became seemingly ‘more capable of judging the external situation, and of making decisions’.90 She observed that ‘[i]n those days when the war seemed inevitable […] the patients became less anxious and seemed to accept the position with a certain amount of resignation and determination, and the wish to help and co-operate had become stronger’.91

As Bion noted, people tended to become less anxiously paralysed and more focused on social cooperation when external reality gave them the opportunity to shift their attention from the uncertain waiting that gave fertile ground for the difficult, unprocessed material of internal phantasy, towards activity. As Klein herself put it, relief was produced by

externalising an internal danger-situation, since a definite knowledge that war was imminent made it less phantastic, and encountering it meant coping with it […] Anxiety was further relieved by the feeling that, by means of this reparation carried out in the outside world, the inner world would be repaired.92

But while Bion was, at this point, concentrated on psychological A.R.P. and the practical ends towards which waiting time might be directed, Klein was more concerned with what an alleviation of an internal anxiety-situation might mean psychoanalytically. A precipitate identification with Chamberlain as a good father seemed for many to be followed by a feeling of numbness, then guilt and shame, as reality suggested that ‘bad parents’ had gained power, both internally and externally; there was ‘a despair of goodness surviving in the internal and external world’.93 Klein similarly inferred that ‘the attitude of bowing to a dictator’ might be another way of evading internal anxiety: a ‘means of denying one’s own hatred of him in order to escape from fears and conflicts the child may feel’.94

By the summer of 1940, just before the Blitz was to bring total war to London, Klein seemed clear, however, that the anxiety produced by external circumstances brought both danger and opportunity. If unprocessed, internal phantasies could lead to psychic catastrophe and death through either a manic evasion of threat (not waiting long enough) or a dangerous paralysis (waiting too long) that signalled a ‘suicidal incapacity to deal with external dangers, and ultimately the means of destroying the dangerous Hitlers inside’.95 As Stonebridge has suggested, here Klein does not simply interpret ‘war anxiety in terms of instinctual anxiety’, as some have claimed; historical conflict is not ‘subsumed by the ahistory of phantasy’.96 But by allowing anxiety about the external war to illuminate an anxious internal reality, and by suspending the ‘war inside’ by bringing it, over time, into analytic understanding, Klein wrote that she saw ‘patients’ courage grow, depression diminish and the capacity to make decisions, etc increase when hatred and guilt connected with early phantasies had been analysed’.97 For Klein, it was thus the capacity of analysis to contain and understand hatred and guilt over time that enabled the patient to hold on to ‘the feeling that goodness cannot be ultimately exterminated’—a feeling ‘based on a better balance between facing danger and yet relying more on internal goodness and trust in some good object’.98 Here, then, Klein describes an analytic fulfilment of the promise for signal anxiety that Freud holds out in 1926: the alarm of anxiety produces a repetition of the past in the anxious present that, through the containment of analytic thinking, enables the possibility that ‘there is yet time’ rather than temporal ‘evacuation’—a time in which courage and belief in the endurance of good objects and a future endures over the psychic catastrophe of an ever-repeated ‘war inside’.

Woolf is clear that being ‘under fire’ halts thinking:

The sound of sawing overhead has increased. All the searchlights are erect. They point at a spot exactly above this roof. At any moment a bomb may fall on this very room. One, two, three, four, five, six … the seconds pass. The bomb did not fall. But during those seconds of suspense all thinking stopped. All feeling, save one dull dread, ceased. A nail fixed the whole being to one hard board. The emotion of fear and of hate is therefore sterile, unfertile.99

The phallic quality of the ‘erect’ searchlight suspends thinking and time in a barren fashion. This state does not last indefinitely, however; instead, there is a subtle shift into another kind of waiting in which memory and a sense of sociality, rather than solitary anxiety or depressed sterility, returns. Directly that fear passes, the mind reaches out and instinctively revives itself by trying to create:

It reaches out to the memory of other Augusts—in Bayreuth, listening to Wagner; in Rome, walking over the Campagna; in London. Friends’ voices come back. Scraps of poetry return. Each of those thoughts, even in memory, was far more positive, reviving, healing and creative than the dull dread made of fear and hate. Therefore if we are to compensate the young man for the loss of his glory and of his gun, we must give him access to the creative feelings. We must make happiness. We must free him from the machine.100

As Kirsty Martin has noted, the injunction to ‘make happiness’ sits oddly in this lyrical passage by seeming to suggest ‘the creation of happiness as a form of national project’.101 But, Martin insists, it has its place in Woolf’s fraught attempts to hold in mind the relationship between profoundly personal states of happiness experienced in the intensity of ‘the moment’ and the demands for the good life of the many that could be marked in sociohistorical time.

Klein, like Woolf and diarist 5278, writes of the need to connect with happiness in the moment in ways that look like a temporary denial of the reality of historical danger: ‘We look at nature, we read a book, we play with a child, we enjoy food, etc, and we have to remind ourselves that our life and country is at stake’.102 But, she writes, if this careful attention to ‘good objects and (belief) in goodness ultimately’ is used not to deny reality but as a commitment to the endurance of these elements, it may, in fact, ‘help us to take steps to preserve goodness externally, and may internally help us to keep calm in the face of danger’.103 The time that this air raid ‘blows into existence’, then, is one of creativity and dependence in which action and violence are somehow suspended, but the possibility of caring for ‘good objects’ in the internal and external world endures. For Woolf, this happiness seems to be a kind of women’s time, just as her account of women’s writing insists on a non-injurious fidelity to ‘the moment’. Women must make happiness in the present. This comes, however, through creative endeavour imagined neither as utterly individualistic nor as completely socialised. Both of these possibilities are refused in the essay in Woolf’s resistance to mortgaging the present to a simple idea of the future of nationalist myths or making children that would merely feed the war machine. Neither explicitly productive nor flagrantly unproductive, this potentially queer fidelity to the complex sociality of the present swells as if gestating a kind of waiting time; it produces a suspended present not of machines or national time regimes but of embodied selves that need tending, that need tenderness. And what this produces, for Woolf, at least for this moment, is a place and time for going on through forms of creative, subjective sociality in which the world is maintained, continued and repaired—it is cared for—even when all going on seems radically foreclosed.

Over time, Bion let go of the idea of psychoanalysis as a grand curative project. Towards the end of a life as scarred and moved to thinking by conflict as the century it spanned, Bion sought to give up waiting *for*, just as Western cultures of modernity became increasingly uncertain of religious ideas of deliverance, positivist narratives of history, or a progressive future into which one could simply step. Instead, he suggested the importance of analytic endurance and waiting *with*—an attitude of presence and patience modelled after Keats’s notion of ‘negative capability’. In his 1973–74 Brazilian lectures, Bion described a model of analytic thinking that resists the defences of intellectualisation and the tendency to get taken up with ‘memory’ (of previous sessions) or future ‘desire’ (for a cure, or interpretation):

Instead of trying to bring a brilliant, intelligent, knowledgeable light to bear on obscure problems, I suggest we bring to bear a diminution of the ‘light’—a penetrating beam of darkness; a reciprocal of the searchlight […] The darkness would be so absolute that it would achieve a luminous absolute vacuum. So that, if any object existed, however faint, it would show up very clearly.104

Here, the beam of darkness becomes a mirror image of the searchlight used in World War II to create artificial moonlight to dazzle and illuminate the enemy. This mode of negative yet containing attention, as analyst and patient wait together, instead becomes a way of doing justice to what exists in the present, including feelings of ‘unthinkable anxiety’, ‘nameless dread’, and acts of psychic violence that annihilate and ‘kill time’. What is evoked instead is a capacity to wait for the anxious bombardment of thoughts to pass and for something else, not necessarily a grand narrative of the future, but a commitment to care and to a time in which good objects will endure—a way of going on when all going on might be purposeless—to emerge in its wake.

## Notes

1. Elizabeth Bowen (1999), “Mysterious Kôr,” *Collected Stories* (London: Vintage), 821–834, 821.

2. Bowen, “Mysterious,” 822.

3. Bowen, “Mysterious,” 823.

4. Elizabeth Bowen (1986), *The Mulberry Tree: Writings of Elizabeth Bowen* (London: Virago), 250.

5. Bowen, “Mysterious,” 823.

6. Max Weber (2009), “Science as a Vocation,” *From Max Weber: Essays in Sociology*, edited by H H Gerth and C Wright Mills (London: Routledge), 129–158, 139.

7. See, Rosalind Williams (2013), *The Triumph of the Human Empire: Verne, Morris and Stevenson at the End of the World* (Chicago: University of Chicago Press).

8. See, David Trotter (2013), *Literature in the First Media Age* (Cambridge: Harvard University Press), 73–82.

9. Elizabeth Bowen (1998), *The Heat of the Day* (London: Vintage), 100.

10. Bowen, *Mulberry Tree*, 98.

11. Bowen, *Mulberry Tree*, 95.

12. Hilda Doolittle (2014), *Within the Walls and What Do I Love?*, edited by Annette Debo (Gainesville: University of Florida Press), 108.

13. See Michael Roper (2009), *The Secret Battle: Emotional Survival in the Great War* (Manchester: Manchester University Press), 86.

14. Paul K Saint-Amour (2015), *Tense Future: Modernism, Total War, Encyclopedic Form* (Oxford: Oxford University Press).

15. Stanley Baldwin (1932), “A Fear for the Future,” Speech to Parliament, November 10, 1932.

16. Michal Shapira (2013), *The War Inside: Psychoanalysis, Total War, and the Making of the Democratic Self in Postwar Britain* (Cambridge: Cambridge University Press), p. 26.

17. Saint-Amour, 13.

18. Saint-Amour, 7, 8.

19. Sigmund Freud (1925–1926), *Inhibitions*, *Symptoms and Anxiety*, *The Standard Edition of the Complete Psychological Works of Sigmund Freud*, Vol. XX, 75–176, 132.

20. Lynsey Stonebridge (2007), *The Writing of Anxiety: Imagining War-time in Mid-Century British Culture* (London: Palgrave), 18–19.

21. Saint-Amour, 18.

22. Stonebridge, *Writing*, 18.

23. Freud, *Inhibitions*, 165. The phrase ‘yet time’ is Strachey’s fairly straight translation of Freud’s ‘noch Zeit’.

24. Bowen, ‘Mysterious’, 821.

25. Lisa Baraitser (2017), *Enduring Time* (London: Bloomsbury).

26. Joan Tronto (2013), *Caring Democracy: Markets, Equality, and Justice* (New York: NYU Press), 19.

27. ‘Concern’ captures the same proximity of worry and looking after. In German, *Sorge* also means both to take care of and to worry.

28. Maria Puig de la Bellacasa (2017), *Matters of Care: Speculative Ethics in More than Human Worlds* (Minneapolis: University of Minnesota Press, 2017), 206.

29. De la Bellacasa, 207.

30. Ghassen Hage (2009), *Waiting* (Melbourne: University of Melbourne Press), 4, 6.

31. Reinhart Koselleck (2004), *Futures Past: On the Semantics of Historical Time*, trans. Keith Tribe (New York: Columbia University Press), 11-17.

32. Benjamin H Snyder (2013), “From Vigilance to Busyness: A Neo-Weberian Approach to Clock Time,” *Sociological Theory*, 31, no. 3, (September 2013), 243–266, 252–253.

33. Koselleck, 21-22.

34. See, Stephen Kern (1983), *The Culture of Time and Space, 1880–1918* (Cambridge: Harvard University Press), 10–35.

35. Henri Bergson (1922), *Creative Evolution*, trans. Arthur Mitchell (London: Macmillan), 8.

36. Bergson, *Creative Evolution*, 9–10.

37. Anson Rabinbach (1992), *The Human Motor: Energy, Fatigue and the Origins of Modernity* (Berkeley: California University Press), 259.

38. Henri Barbusse (2003), *Under Fire*, trans. by Robin Buss (London: Penguin), 18.

39. Kate McLoughlin (2011), *Authoring War: The Literary Representation of War from the Iliad to Iraq* (Cambridge: Cambridge University Press), p. 110–134.

40. Eugène Minkowski (1970), *Lived Time: Phenomenological and Psychological Studies*, trans. Nancy Metzel (Evanston: Northwestern University Press), 69.

41. Minkowski, 87–88.

42. Minkowski, 332.

43. Minkowski, 298.

44. Minkowski, 299.

45. Bowen, “Mysterious,” 834.

46. Thomas S Davis (2016) wonders if ‘Myserious Kôr’ ends on a note of ‘imperial nostalgia’ in *The Extinct Scene: Late Modernism and Everyday Life* (New York: Columbia University Press), 185. For an account of how ‘timelessness’ frequently reproduces White racial privilege, see Charles W Mills (2014), ‘White Time: The Chronic Injustice of Ideal Theory,’ *Du Bois Review*, 11, 1 (January, 2014), pp. 27–42.

47. Lara Feigel (2014), *The Love-charm of Bombs: Restless Lives in the Second World War* (London: Bloomsbury), 24.

48. Barbusse, 18.

49. Elizabeth Freeman (2010), *Time Binds: Queer Temporalities, Queer Histories* (Durham and London: Duke University Press), x.

50. Freeman, xii.

51. Mary Borden (2008), *The Forbidden Zone* (London: Hesperus Press), 101–102.

52. Borden, 102.

53. Borden, 103–104.

54. Borden, 104.

55. Bowen, *Mulberry*, 97.

56. See, Hugh Kenner (1973), *A Reader’s Guide to Samuel Beckett* (London: Thames and Hudson); Marjorie Perloff (2005), “In Love With Hiding: Samuel Beckett’s War,” *The Iowa Review*, 35, no. 1: 76-103, pp. 81–2.

57. Samuel Beckett (2012), *Endgame*, *Complete Dramatic Works* (London: Faber and Faber).

58. See, for example, James Knowlson (1996), *Damned to Fame: The Life of Samuel Beckett* (London: Bloomsbury), 175–186; Laura Salisbury (2011), “Bulimic Beckett: Food for Thought and the Archive of Analysis,” *Critical Quarterly*, 53, no. 3: 61-80.

59. Beckett to McGreevy, quoted in Knowlson, 176.

60. W. R Bion (1940), “The ‘War of Nerves’: Civilian Reaction, Morale and Prophylaxis,” *The Neuroses in War*, edited by Emanuel Miller (London: Macmillan), 180–200, 183. He borrows his title from a ‘modern bard’, likely Frederic Prokosch, whose poem ‘War of Nerves’ lists a chaotic concatenation of primitive psychic states that either overwhelm their subjects, as ‘mad wheels are turning faster and faster’, or are evaded through gestures, technologies, and practices of modernity that overly stimulate and anaesthetise: ‘Marconi, Marx, Miss Garbo, Dr. Goebbels and Dr. Freud/ Huddle like assassins over the staggering void’. Frederic Prokosch (1940), *Death at Sea* (London: Chatto and Windus), 35, 36.

61. See Edward Glover (1941), “Notes on the Psychological Effects of War Conditions on the Civilian Population (1),” *International Journal of Psycho-Analysis*, 22, pp. 132–1. For further details of the committee’s work, see L Stonebridge (1998), “Anxiety at a Time of Crisis,” *History Workshop Journal*, no. 45 (Spring), pp. 170–182, 172.

62. Bion, “War,” 181.

63. Bion, “War,” 195.

64. Bion, “War,” 189.

65. W. R Bion (1984), “A Theory of Thinking,” *Second Thoughts* (London: Karnac), p. 116.

66. W. R Bion (1991), *The Long Week-end, 1897–1919: Part of a Life* (London: Karnac), 117.

67. Bion, *Long Week-end,* 120.

68. Bion, *Long Week-end*, 132.

69. W. R Bion (1997), *War Memoirs: 1917–19*, edited by Francesca Bion (London: Karnac), 255. Kay M Souter (2009) writes revealingly of Bion’s traumatised return to this incident across his writing in “The ‘War Memoirs’: Some Origins of the Thought of W. R. Bion,” *International Journal of Psychoanalysis*, 90, 795–808.

70. Bion, *War Memoirs*, 122.

71. Mass Observation was founded in 1937 with the aim of recording everyday life in Britain via a panel of around 500 untrained volunteer observers who either maintained diaries or replied to open-ended questionnaires.

72. Diarist 5278 (1940), August 1940, http://www.massobservation.amdigital.co.uk/Documents/Images/Diarist-5278/68.

73. Bowen, *Mulberry*, 95.

74. Diarist 5278, http://www.massobservation.amdigital.co.uk/Documents/Images/Diarist-5278/69.

75. Stonebridge, ‘Anxiety,’ 172.

76. Edgar Jones (2012), “’The gut war’: Functional Somatic Disorders in the UK during the Second World War,” *History of the Human Sciences*, 25, no. 5 (December 2012), 30–48.

77. W. R Bion (1961), *Experiences in Groups and Other Papers* (London: Tavistock, 1961), 25.

78. Bion, *Experiences*, 15–16.

79. Bion, *Experiences*, 154.

80. Bion, *Experiences*, 153.

81. Daniel Pick (2014), *The Pursuit of the Nazi Mind: Hitler, Hess, and the Analysts* (Oxford: Oxford University Press), 199.

82. Bion, *Experiences*, 158.

83. Bion, “Thinking,” 111.

84. Bion, “Thinking,” 113.

85. Bion, “Thinking,” 117.

86. Virginia Woolf (1940), “Thoughts on Peace in an Air Raid,” *The New Republic*, October 21, 1940. https://newrepublic.com/article/113653/thoughts-peace-air-raid.

87. Melanie Klein (2018), “What does death represent to the individual?” *Melanie Klein Archive*, Wellcome Library, PPKLE/C/96, 2.

88. Melanie Klein (1938), “Crisis I: Reactions of Patients to Events in Austria. Patients ABCDEF,” *Melanie Klein Archive*, Wellcome Library, PPKLE/B/83/84, 1.

89. Melanie Klein (1938), “Crisis II: Before and After the Crisis. Patients A, C, MI, MII, R, SI, SII, T,” *Melanie Klein Archive*, Wellcome Library, PP/KLE/B.85:Box AMS/MF/243, 101.

90. Klein, “Crisis II,” 101.

91. Klein, “Crisis II,” 102.

92. Klein, “Crisis II,” 125.

93. Klein, “Crisis II,” 116.

94. Klein, “Crisis II,” 109.

95. Klein, “What,” 3.

96. Stonebridge, “Anxiety,” 178.

97. Klein, “What,” 3.

98. Klein, “What,” 6.

99. Woolf, https://newrepublic.com/article/113653/thoughts-peace-air-raid.

100. Woolf, https://newrepublic.com/article/113653/thoughts-peace-air-raid.

101. K. Martin (2014), “Virginia Woolf's Happiness,” *Essays in Criticism: a Quarterly Journal of Literary Criticism*, 64, no. 4, 394–414.

102. Klein, “What,” 6.

103. Klein, “What,” 7.

104. W. R Bion (1990), Brazili*an Lectures* (London: Karnac), 21–22.
